# Characterization, sequencing and comparative genomic analysis of vB_AbaM-IME-AB2, a novel lytic bacteriophage that infects multidrug-resistant *Acinetobacter baumannii* clinical isolates

**DOI:** 10.1186/1471-2180-14-181

**Published:** 2014-07-05

**Authors:** Fan Peng, Zhiqiang Mi, Yong Huang, Xin Yuan, Wenkai Niu, Yahui Wang, Yuhui Hua, Huahao Fan, Changqing Bai, Yigang Tong

**Affiliations:** 1 State Key Laboratory of Pathogen and Biosecurity, Beijing Institute of Microbiology and Epidemiology, Beijing 100071, China; 2 Department of Respiratory Medicine, PLA Hospital 307, Beijing 100071, China; 3 The Third Xiangya Hospital of Central South University, Changsha 410013, China

**Keywords:** *Acinetobacter baumannii*, Bacteriophage, Characteristics, Genome

## Abstract

**Background:**

With the use of broad-spectrum antibiotics, immunosuppressive drugs, and glucocorticoids, multidrug-resistant *Acinetobacter baumannii* (MDR-AB) has become a major nosocomial pathogen species. The recent renaissance of bacteriophage therapy may provide new treatment strategies for combatting drug-resistant bacterial infections. In this study, we isolated a lytic bacteriophage vB_AbaM-IME-AB2 has a short latent period and a small burst size, which clear its host’s suspension quickly, was selected for characterization and a complete genomic comparative study.

**Results:**

The isolated bacteriophage vB_AbaM-IME-AB2 has an icosahedral head and displays morphology resembling *Myoviridae* family. Gel separation assays showed that the phage particle contains at least nine protein bands with molecular weights ranging 15–100 kDa. vB_AbaM-IME-AB2 could adsorb its host cells in 9 min with an adsorption rate more than 99% and showed a short latent period (20 min) and a small burst size (62 pfu/cell). It could form clear plaques in the double-layer assay and clear its host’s suspension in just 4 hours. Whole genome of vB_AbaM-IME-AB2 was sequenced and annotated and the results showed that its genome is a double-stranded DNA molecule consisting of 43,665 nucleotides. The genome has a G + C content of 37.5% and 82 putative coding sequences (CDSs). We compared the characteristics and complete genome sequence of all known *Acinetobacter baumannii* bacteriophages. There are only three that have been sequenced *Acinetobacter baumannii* phages AB1, AP22, and phiAC-1, which have a relatively high similarity and own a coverage of 65%, 50%, 8% respectively when compared with our phage vB_AbaM-IME-AB2. A nucleotide alignment of the four *Acinetobacter baumannii* phages showed that some CDSs are similar, with no significant rearrangements observed. Yet some sections of these strains of phage are nonhomologous.

**Conclusion:**

vB_AbaM-IME-AB2 was a novel and unique *A. baumannii* bacteriophage. These findings suggest a common ancestry and microbial diversity and evolution. A clear understanding of its characteristics and genes is conducive to the treatment of multidrug-resistant *A. baumannii* in the future.

## Background

*Acinetobacter baumanni* is a non-fermentative, aerobic, gram-negative bacillus, and is an opportunistic pathogen with global distribution. It is frequently found in elderly patients and cancer patients with compromised immune function, especially in intensive care units. With the use of broad-spectrum antibiotics, immunosuppressive drugs, and glucocorticoids, *A. baumannii* (AB) has become a major nosocomial pathogen species [1]. Multidrug-resistant (MDR), extensively drug-resistant (XDR), and pan drug-resistant (PDR) *A. baumannii* strains are increasingly prevalent [2]. MDR-AB refers to *A. baumannii* strains that are resistant to at least three of the following five types of antimicrobial agents: cephalosporins, carbapenems, β-lactamase inhibitors (including piperacillin/tazobactam, cefoperazone/sulbactam, ampicillin/sulbactam), fluoroquinolones, and aminoglycosides [2-4].

Bacteriophage therapy is a potential alternative treatment for multidrug-resistant bacterial infections [5]. A bacteriophage is a bacterial virus that can lyse and kill the host cell. Phage-related studies have gone through three stages. Félix d’Herelle discovered bacteriophage for the treatment of bacterial infections in 1917 [6]. After the emergence of antibiotics in the 1940s, phages were seldom used for therapeutic purposes, and mainly functioned as molecular and genetic research tools. With the recent emergence of multidrug-resistant bacteria, however, there has been renewed interest in methods of phage therapy [7]. In this study we isolated a lytic bacteriophage IME-AB2, and compared biological characteristics and genomic sequence with other *Acinetobacter baumannii* phages. The genomes of *A. baumannii* phages IME-AB2, *A. baumannii* AB1, *A. baumannii* AP22, and A. *baumannii* phiAC-1 were compared thoroughly in this study. To our knowledge this is the first report of comparison of the characteristics and complete genome sequence of *Acinetobacter baumannii* bacteriophages. A clear understanding of its genes is conducive to the treatment of multidrug-resistant *A. baumannii* in the future.

## Results

### Isolation of a lytic bacteriophage against multidrug-resistant *A. baumannii*

**Figure 1:**
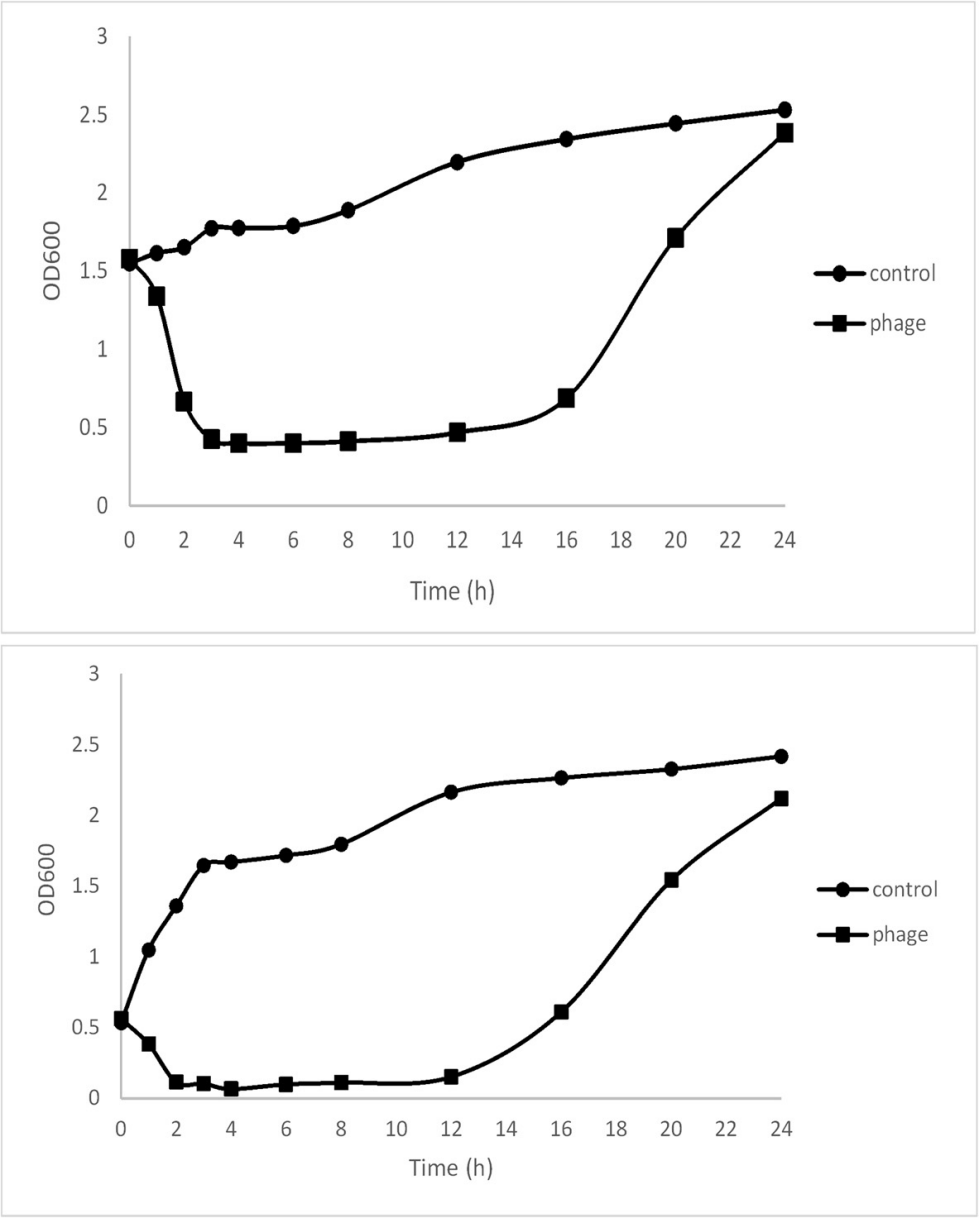
**The MDR-AB2 suspension at the different optical density (OD600nm) reached to 0.4 from 1.6 and reached to 0.08 from 0.6 respectively after added 200ul IME-AB2 (1 × 10**^**11 **^**pfu/ml) to the 10 ml MDR-AB2 suspension.** It clear its host’s suspension in just 4 hours. The control shows increasing OD600nm. The MDR-AB2 suspension added with IME-AB2 finally became turbid in 24 hours.

**Figure 2:**
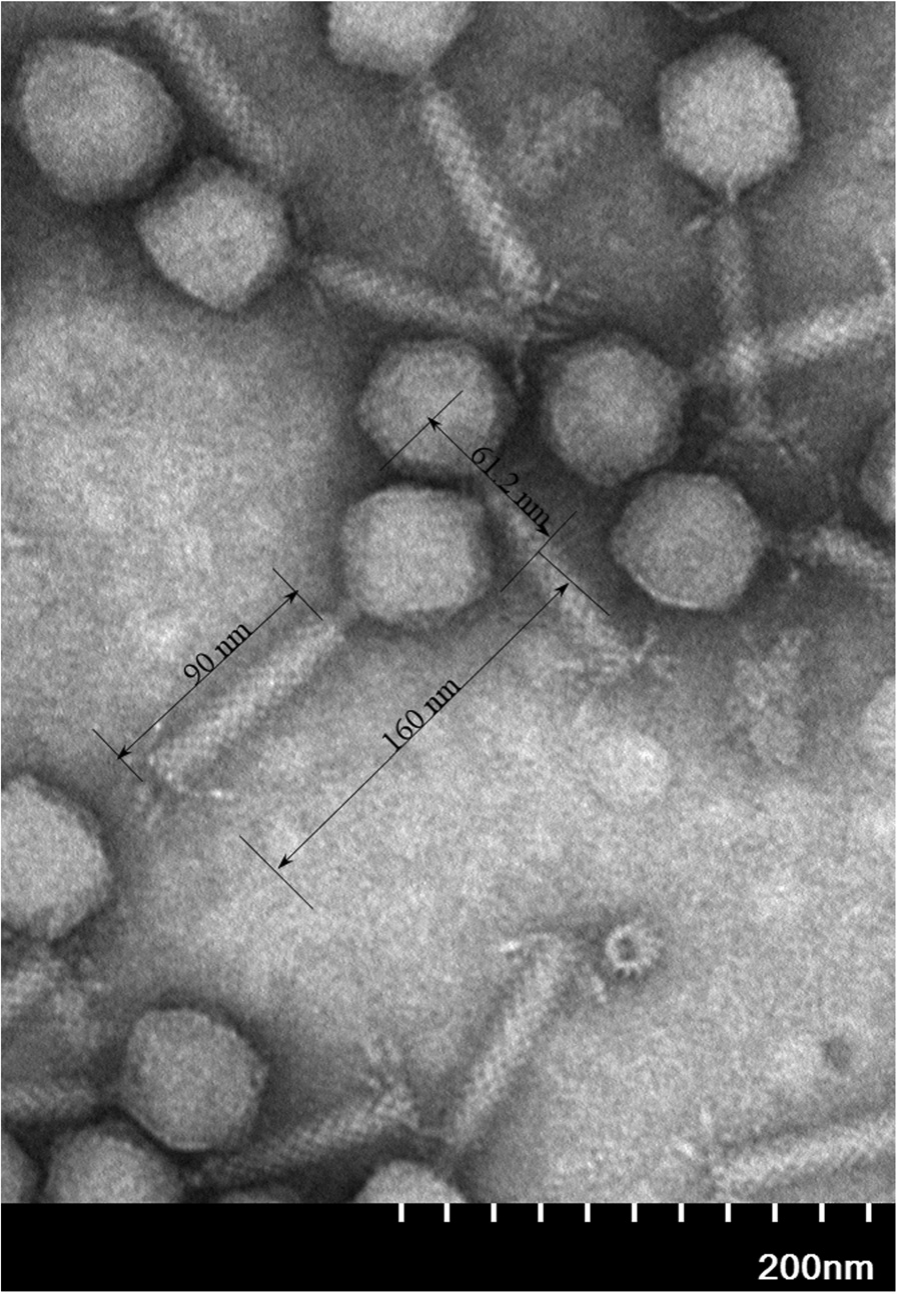
**Transmission electron microscopy of phage IME-AB2.** The bidirectional arrows indicated the length of intact phage, phage tail and head. The bar represents a length of 200 nm.

*A. baumannii* strain MDR-AB2, isolated from a sputum sample of a patient with pneumonia at PLA Hospital 307, was resistant to multiple antibiotics (Table 1). The bacteria was used to screen bacteriophages in sewage samples from PLA Hospital 307. The isolated phage was designated as vB_AbaM-IME-AB2 following the recommendation by International Committee on Taxonomy of Viruses in phage nomenclature [8]. The pahge IME-AB2 could form clear plaques in the double-layer assay and clear its host’s suspension in just 4 hours (Figure 1), indicating that it is a lytic phage. In order to check the development of resistance, we had extended the period of the experiment to 24 h. The result indicated that the bacterial suspension became turbid finally. The final suspension was plated on solid LB culture and then some single bacterial clones were picked to be used for 16 s rDNA sequencing. The sequences of 16 s rDNA proved that the final suspension was *A. baumannii* that developed resistance to IME-AB2. The phage particles were concentrated with PEG6000 and then purified with a cesium chloride gradients density to a titer of 1 × 10^11^ pfu/ml. Observation under an electron microscope showed that the phage IME-AB2 consisted of an icosahedral head and a contractile tail. The total length of the phage from the top of the head to the bottom of the tail was about 160 nm, with the head measuring approximately 61.2 nm, and the tail about 90 nm. This morphology suggested that phage IME-AB2 should be classified as a member of the *Myoviridae* family (Figure. 2). Among the 22 clinical strains of *A. baumannii*, only three strains of *A. baumannii* (MDR-AB1139, MDR-AB2 and MDR-AB11) could be lysed by the phage IME-AB2.

**Table 1:** **Antibiotic resistance profile of *A. baumannii *** **strain MDR-AB2**

**Antibiotics**	**MIC (μg/ml)**	**Sensitivity**	**Antibiotics**	**MIC (μg/ml)**	**Sensitivity**
Ampicillin	≥ 32	Resistant	nitrofurantoin	≥ 512	Resistant
Ciprofloxacin	≥ 4	Resistant	ampicillin/sulbactam	≥ 32	Resistant
Gentamicin	≥ 16	Resistant	aztreonam	≥ 64	Resistant
Imipenem	≥ 16	Resistant	cefepime	≥ 64	Resistant
Meropenem	≥ 16	Resistant	cefotetan	≥ 64	Resistant
Piperacillin	≥ 128	Resistant	ceftazidime	≥ 64	Resistant
Piperacillin/tazobactam	≥ 128	Resistant	ceftriaxone	≥ 64	Resistant
Tobramycin	≥ 16	Resistant	cefuroxime axetil	≥ 64	Resistant
Cotrimoxazole	≥ 320	Resistant	cefuroxime sodium	≥ 64	Resistant
Levofloxacin	≥ 8	Resistant	cefoperazone/sulbactam	≥ 64	Resistant

### Growth and lytic characteristics of IME-AB2

**Figure 3:**
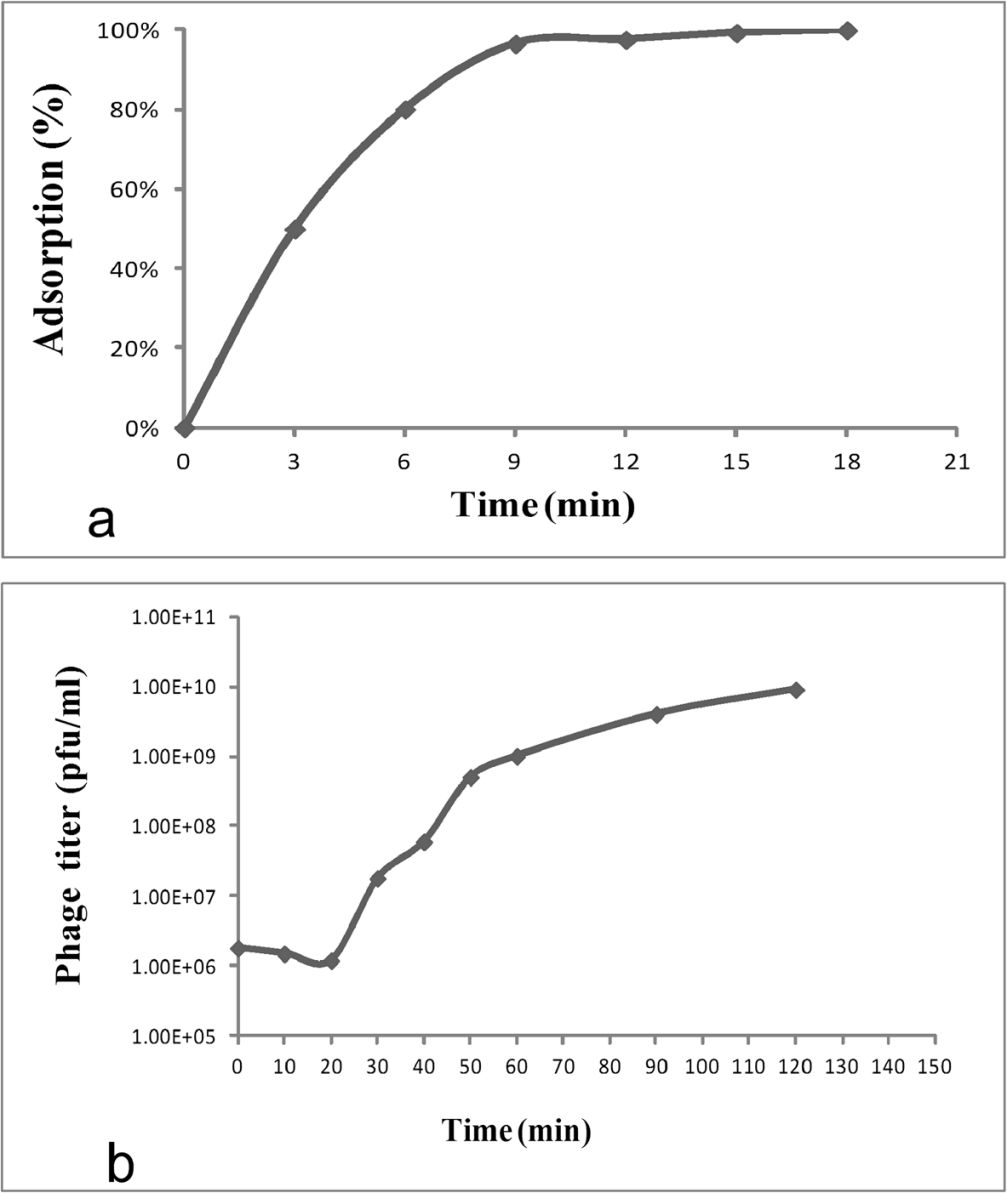
**Biological characteristics of phage IME-AB2. a**. Host adsorption ability of phage IME-AB2. **b**. One-step growth curve of phage IME-AB2.

To determine the optimal multiplicity of infection (MOI) of IME-AB2, the phage and its host cells were mixed at various ratios, and incubated for 3.5 h at 37°C. The results indicated that a MOI of 20 gave the highest production of phage progeny (3.5 × 10^11^ pfu/ml). To examine the host adsorption ability of phage IME-AB2, host bacteria were infected with IME-AB2 at a MOI of 0.1 and incubated at 37°C. Aliquots were taken at 0, 3, 6, 9, 12, 15, and 18 min post-infection and assayed for the absorbed phage by titration using the double-layer method. The percentages of phage absorption at different time points were plotted (Figure 3a). The results showed that phage IME-AB2 had an adsorption rate of 50% within 3 min, 80% within 6 min and 99% within 9 min.

For one-step growth curve analysis, MDR-AB2 cells (OD600 = 0.3) were infected with phage IME-AB2 at a MOI of 0.1. The bacteriophage was allowed to adsorb for 15 min at 37°C [9]. The mixture was then centrifuged at 12,000 × g for 30 s to remove unadsorbed phage particles, and the resultant pellet was re-suspended in 5 ml of LB medium. Samples were incubated at 37°C and collected every 10 min during 0–60 min, as well as at 90 and 120 min [10]. As shown in Figure 3b, the latent period of phage IME-AB2 lasted for 20 min, the burst period reached a peak at 30 min, and the phage multiplication reached the final plateau phase at 50 min. The burst size of phage IME-AB2 was determined to be 62 pfu/cell (burst size = the total number of phages liberated at the end of one cycle of growth /the number of infected bacteria) [11].

### Analysis of the phage proteins and genome

**Figure 4:**
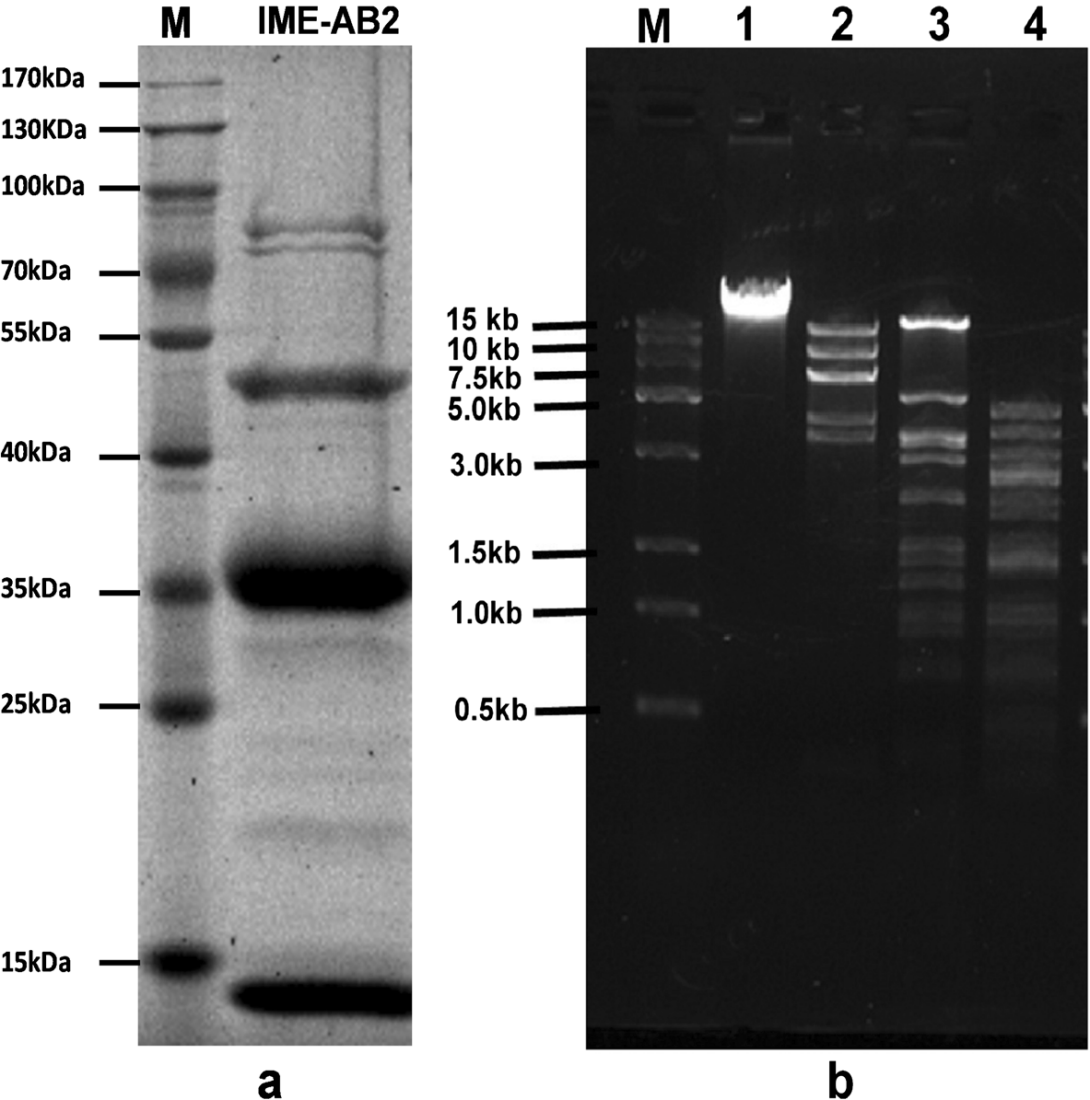
**Protein and genomic DNA analysis of phage IME-AB2. a**. SDS-PAGE gel (10%) of whole protein from phage IME-AB2. Molecular weights of protein marker was indicated by lines. **b**. Endonuclease digestion analysis of phage IME-AB2 genomic DNA. Phage IME-AB2 genomic DNA was digested with the restriction enzyme *Nde*I, *HincII* and *HindIII*. The digested DNA fragments were separated by 1% agarose gel electrophoresis. M, DNA molecular weight marker; Lanes 1, undigested phage IME-AB2 genomic DNA; lane 2, 3, 4, genomic DNA digested with *NdeI* , *HincII* and *HindIII*, respectively.

Purified phage particles were denatured in loading buffer (50 mM Tris–HCl, 2% Sodium Dodecyl Sulfate-polyacrylamide, 0.1%Bromophenol blue, 10% Glycerol and 1% β-Mercaptoethanol) and heated in a boiling water bath for 5 min, followed by separation of the proteins by sodium dodecyl sulfate-polyacrylamide gel electrophoresis (SDS-PAGE). The results indicated that the structural proteins of phage IME-AB2 showed a pattern of nine protein bands in 10% SDS-PAGE gel, with molecular masses ranging from 15–100 kDa (Figure 4a). The most abundant protein band in the gel above 35 kDa was analyzed with liquid sampling Mass Spectrometry (LS-MS) and proved to be the phage putative capsid protein.

The genome analysis indicated that phage IME-AB2 has a double-stranded DNA genome, approximately 40 kb in size. The genome of phage IME-AB2 could be digested with endonuclease *Nde*I, *HincII* and *HindIII* (Figure 4b). It was found that endonuclease enzymes, *HindIII* and *HincII*, have the 35 and 16 cutting sites on the genome of phage IME-AB2 respectively by Vector NTI [12]. Compared to other *A. baumannii* complete genome , the two endonucleases also have most restriction enzyme cutting sites on them.

**Figure 5:**
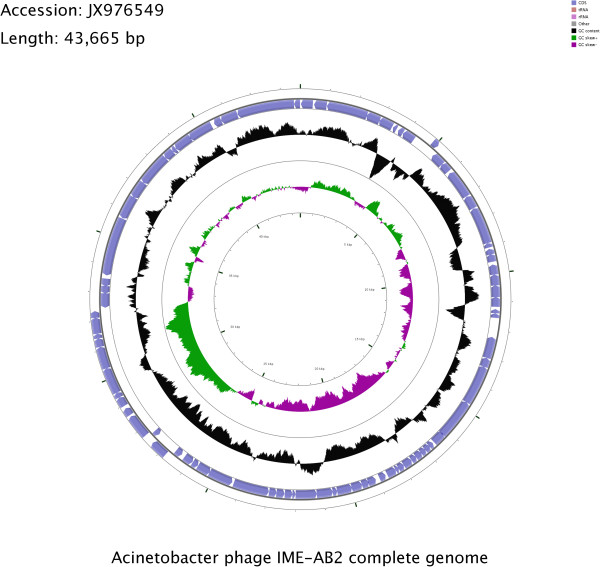
**Circular map of the IME-AB2 genome prepared using CGView.** The outer ring denotes the IME-AB2 genome and CDSs. The inner rings show G + C content and G + C skew, where peaks represent the positive (outward) and negative (inward) deviation from the mean G + C content and G + C skew, respectively.

**Figure 6:**
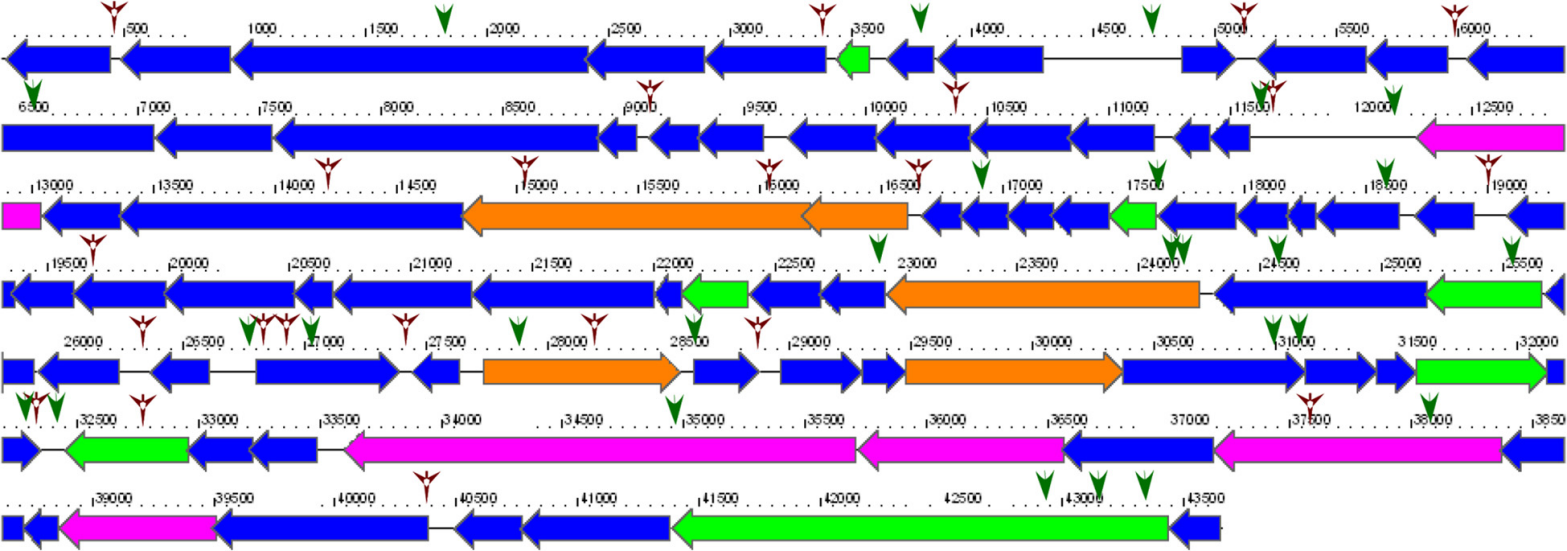
**Genome map of phage IME-AB2.** Arrows indicate putative CDSs, along with their orientations. Functionally assigned genes are differently colored (purple, structural gene; green, metabolic gene; orange, replication/packaging-associated gene; blue, other gene). Promoters are illustrated as green arrowheads, while terminators are displayed as red arrowheads.

**Figure 7:**
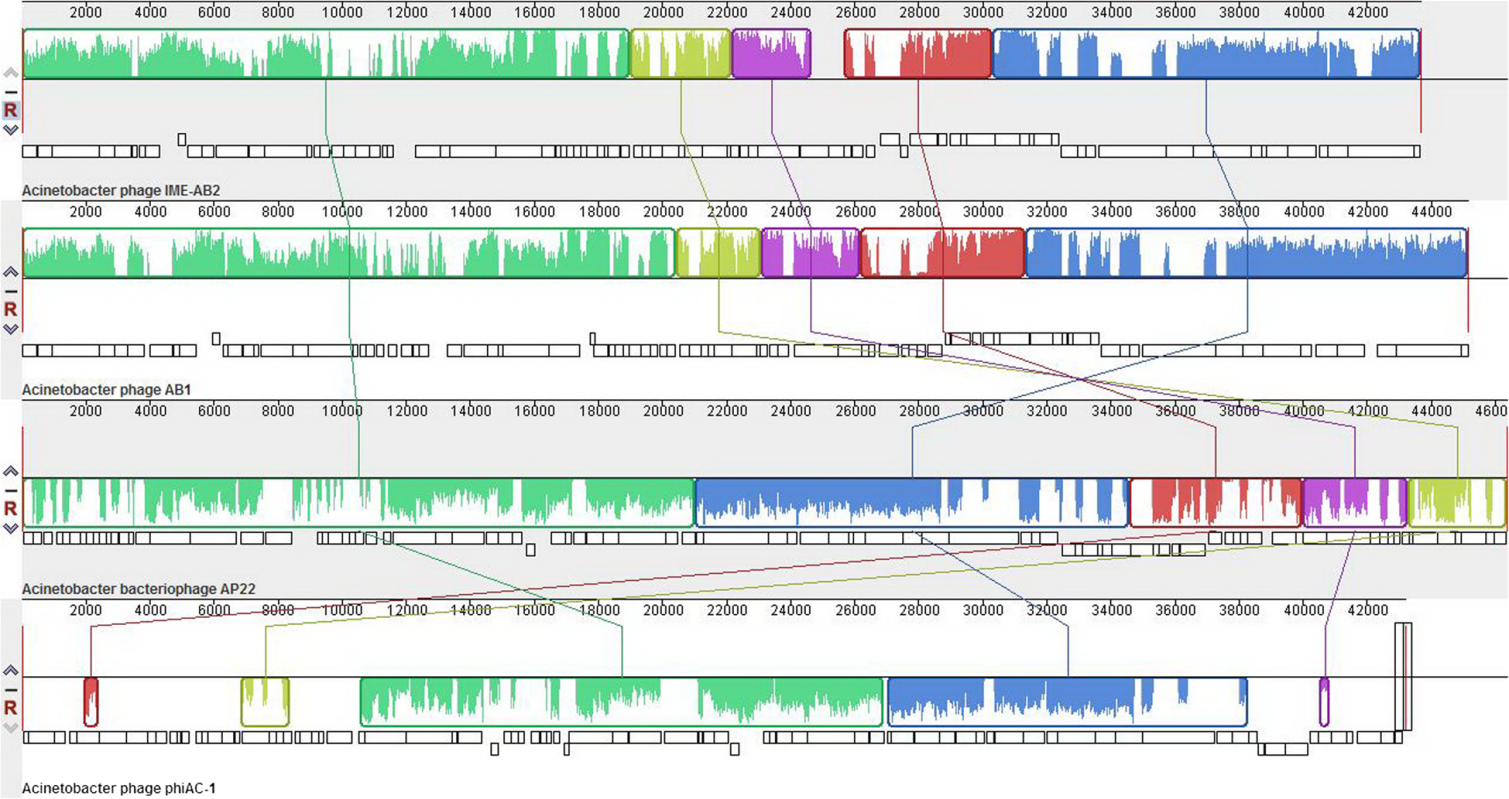
**Multiple genome alignment performed using Mauve software (**http://asap.ahabs.wisc.edu/mauve/**) and the chromosomes of *A. baumannii *IME-AB2, *A. baumannii *AB1, *A. baumannii *AP22, and A. *baumannii *phiAC-1. IME-AB2 is the reference for alignments and comparisons to the three other strains.** Boxes with identical colors represent local colinear blocks (LCB), indicating homologous DNA regions shared by two or more chromosomes without sequence rearrangements. LCBs indicated below the horizontal black line represent reverse complements of the reference LCB.

High-throughput sequencing of the phage genomic DNA generated 311,503 valid reads with which the complete sequence of the genome was assembled using both Velvet and CLC Genomic Workbench, with an average coverage of 785 × [13]. The complete genome of phage IME-AB2 consists of 43,665 bp, with an average GC content of 37.5% (Figure 5). Annotation results showed that the genome encodes 82 coding sequences (CDSs) (GenBank Assession number: JX976549). The classification of the 82 CDSs is shown in Table 2 and Figure 6. The complete genome of IME-AB2 is organized into three functional units which encoding structural proteins, metabolic proteins and packaging-associated proteins respectively. No tRNA was found in the genome of IME-AB2, and no significant proteins considered to be markers of temperate bacteriophages were identified. Running blastn showed that the isolated IME-AB2 has a high similarity to *Acinetobacter* phage AB1 (Genbank Accession Number: HM368260.1), *Acinetobacter* phage AP22 (Genbank Accession Number: HE806280.1) and *Acinetobacter* phage phiAC-1 (GenBank accession number: JX560521), which were isolated in China, Russia and Korea respectively. The phage AB1, AP22 or phiAC-1 has a genome of about 45 kb and owns a coverage of 65%, 50%, 8% respectively when compared with the isolated phage IME-AB2. Genomic annotation found that IME-AB2 encodes 82 CDSs, AB1 85 CDSs, AP22 89 CDSs, phiAC-1 82 CDSs. The 82 CDSs from IME-AB2 shared 63 homologues with AB1, 60 homologues with AP22 and 36 homologues with phiAC-1 respectively (Table 3). Totally, 22 of the 82 CDSs encoded by IME-AB2 were identified to be putatively functional. Genomic analysis revealed that the bacteriophage IME-AB2 was most closely related to AB1. A nucleotide alignment of the four *Acinetobacte*r *baumannii* phages showed that some functional regions are highly homologous, with no significant rearrangements observed (Table 3 and Figure 7). It revealed a stable area. Stability is suggested from the high level of nucleotide identity, lack of inversions and other major rearrangements, and the stabilizing selection inferred for virtually all genes harboring synonymous and non-synonymous mutations [14]. Functional related genes are sequential, yet there are a lot of breakpoint modules obviously and some sections of these strains of phages are nonhomologous (Table 3 and Figure 7). It illustrated that these structural genes had occurred in the extensive structural rearrangements during evolution. Bacteriophages are the most diverse and abundant biological entities in nature environment. Most of them can hardly to be found homologous to another bacteriophage, which means evolutionary success obtained by bacteriophages. Furthermore, the diversity is such that even genes with required functions cannot always be recognized. During bacteriophage evolution,the elimination or recombination of genes result in the diversity and meanwhile confer a selective advantage to survive and infect so that phages can better adapt to host bacteria.

**Table 2:** Functional classification of the 82 CDSs in the IME-AB2 genome

**Category**	**CDSs and putative functions**
Structural proteins	CDS.12 putative capsid protein.
	CDS.13 putative structural protein.
	CDS.24 putative phage head protein.
	CDS.71 putative tail fiber.
	CDS.72 similar to the N-terminal region of tail fiber protein.
	CDS.74 putative baseplate J-like protein.
	CDS.77 putative phage baseplate assembly protein.
Metabolic proteins	CDS.06 putative cobalt transport protein.
	CDS.08 putative RNA polymerase.
	CDS.33 putative binding HTH domain or homeodomain-like.
	CDS.47 putative bacteriophage-associated immunity protein.
	CDS.52 putative HNH endonuclease domain protein.
	CDS.66 putative nucleoside triphosphate pyrophosphohydrolase.
	CDS.68 putative lysozyme family protein.
	CDS.81 putative lysozyme protein.
Replication/packaging-associated proteins	CDS.26 putative phage head portal protein.
	CDS.27 putative phage terminase, large subunit.
	CDS.28 putative phage terminase,small subunit.
	CDS.50 putative replicative DNA helicase.
	CDS.51 putative primosomal protein.
	CDS.58 putative transcriptional regulator.
	CDS.62 putative recombinational DNA repair protein.
Other hypothetical proteins	CDS.1,2,3,4,5,7,8,9,10,11,12,13,14,15,16,17,18,19,20,21,22,23,25,26,29,30,31,
	32,34,35,36,37,38,39,40,41,42,43,44,45,46,47,48,49,51,53,54,55,56,57,59,60,
	61,63,64,65,67,69,70,73,75,76,78,79,80,82.

**Table 3:** **Comparative genomic analysis of *A. baumannii *** **phage IME-AB2, *A. baumannii *** **phage AB1, *A. baumanni i*** **phage AP22, and *A3 baumannii *** **phage phiAC-1 **

**IME-AB2 (82CDSs)**	**AB1 (85CDSs)**	**Score (bits)**	**E-value**	**phiAC-1 (82CDSs)**	**Score (bits)**	**E-value**	**AP22 (89CDSs)**	**Score (bits)**	**E-value**
**CDS.01**	gp01	134	9e-35	0055	190	1e-51	gp42	290	1e-81
**CDS.02**	gp02	192	4e-52	0054	205	3e-56	gp41	130	9e-34
**CDS.03**	gp03	895	0.0	0053	645	0.0	gp40	813	0.0
**CDS.04**	gp04	334	5e-95	0052	207	1e-56	gp39	325	4e-92
**CDS.05**	gp06	345	2e-98	0051	220	1e-60	gp38	329	2e-93
**CDS.06**									
**CDS.07**	gp08	57	8e-12	0050	45	3e-08	gp36	52	3e-10
**CDS.08**	gp09	270	7e-76	0049	99	3e-24	gp35	262	3e-73
**CDS.09**	gp10	150	4e-40	0048	128	2e-33	gp34	150	4e-40
**CDS.10**	gp12	298	3e-84	0047	221	5e-61	gp32	299	2e-84
**CDS.11**	gp13	74	1e-16	0046	75	6e-17	gp31	65	4e-14
**CDS.12**	gp15	207	2e-56	0043	208	1e-56	gp30	250	2e-69
**CDS.13**	gp16	120	2e-30	0042	124	1e-31	gp29	99	6e-24
**CDS.14**	gp17	523	e-151	0041	402	e-115	gp28	546	e-158
**CDS.15**									
**CDS.16**	gp19	144	4e-38	0038	31	5e-04	gp27	36	1e-05
**CDS.17**									
**CDS.18**									
**CDS.19**									
**CDS.20**									
**CDS.21**	gp23	55	4e-11				gp22	71	9e-16
**CDS.22**									
**CDS.23**				0022	40	1e-06	gp21	39	2e-06
**CDS.24**	gp27	414	e-119	0031	325	6e-92	gp18	404	e-116
**CDS.25**	gp28	204	4e-56						
**CDS.26**	gp30	831	0.0	0029	658	0.0	gp17	804	0.0
**CDS.27**	gp31	627	0.0	0028	64	7e-13	gp16	60	8e-12
**CDS.28**	gp33	305	2e-86	0027	22	0.82	gp15	20	2.7
**CDS.29**	gp34	31	6e-04	0038	35	3e-05	gp27	32	3e-04
**CDS.30**	gp34	35	2e-05				gp14	30	6e-04
**CDS.31**	gp34	31	6e-04						
**CDS.32**	gp35	149	1e-39				gp12	83	1e-19
**CDS.33**	gp36	112	1e-28				gp10	114	4e-29
**CDS.34**									
**CDS.35**	gp39	133	6e-35				gp05	137	4e-36
**CDS.36**									
**CDS.37**									
**CDS.38**	gp40	147	3e-39	0010	50	1e-09	gp02	124	3e-32
**CDS.39**	gp42	186	1e-50	0021	152	1e-40			
**CDS.40**							gp88	139	8e-37
**CDS.41**	gp43	92	3e-22						
**CDS.42**	gp44	152	5e-40				gp87	71	1e-15
**CDS.43**	gp45	97	7e-24				gp86	95	2e-23
**CDS.44**	gp46	379	e-108				gp85	390	e-112
**CDS.45**	gp47	388	e-111				gp84	186	6e-50
**CDS.46**	gp48	68	5e-15				gp83	70	9e-16
**CDS.47**	gp49	72	2e-16	0019	42	2e-07	gp82	71	4e-16
**CDS.48**	gp50	192	1e-52						
**CDS.49**	gp51	126	8e-33	0009	42	2e-07	gp79	159	9e-43
**CDS.50**	gp52	863	0.0				gp77	619	e-180
**CDS.51**	gp53	204	2e-55				gp76	157	2e-41
**CDS.52**									
**CDS.53**	gp54	130	5e-34				gp75	133	7e-35
**CDS.54**									
**CDS.55**	gp57	150	5e-40				gp72	128	3e-33
**CDS.56**									
**CDS.57**	gp60	72	3e-16				gp69	74	8e-17
**CDS.58**	gp62	216	6e-59				gp68	295	4e-83
**CDS.59**	gp63	153	6e-41						
**CDS.60**	gp64	187	9e-51				gp66	192	2e-52
**CDS.61**							gp65	123	8e-32
**CDS.62**	gp66	594	e-173						
**CDS.63**	gp67	489	e-141				gp63	38	2e-05
**CDS.64**	gp68	189	2e-51				gp62	187	5e-51
**CDS.65**	gp70	59	2e-12				gp61	60	8e-13
**CDS.66**	gp71	125	5e-32						
**CDS.67**	gp72	143	5e-38	0079	80	1e-18	gp59	128	2e-33
**CDS.68**									
**CDS.69**	gp74	171	3.00E-46				gp56	174	4e-47
**CDS.70**	gp75	175	2e-47				gp55	174	3e-47
**CDS.71**	gp76	221	4e-60	0069	189	1e-50	gp54	252	1e-69
**CDS.72**	gp77	304	1e-85	0068	216	3e-59	gp53	313	2e-88
**CDS.73**	gp78	418	e-120	0067	332	3e-94	gp52	421	e-121
**CDS.74**	gp79	790	0.0	0066	585	e-170	gp51	796	0.0
**CDS.75**	gp80	213	1e-58	0065	164	4e-44	gp50	214	6e-59
**CDS.76**							gp49	59	2e-12
**CDS.77**	gp81	429	e-123	0064	200	2e-54	gp48	430	e-123
**CDS.78**	gp82	575	e-167	0063	437	e-125	gp47	573	e-166
**CDS.79**				0061	124	4e-32	gp46	170	5e-46
**CDS.80**	gp83	352	e-100	0060	277	1e-77	gp45	351	e-100
**CDS.81**	gp84	710	0.0	0057	368	e-104	gp44	715	0.0
**CDS.82**	gp85	77	6e-18	0056	61	6e-13	gp43	145	2e-38

IME-AB2 is the reference for alignments and comparisons to the three other strains (AB1,phiAC-1 and AP22).

### Structural proteins

Seven CDSs encoding structural proteins were identified in the phage IME-AB2. The putative capsid protein (CDS.12) is similar to that of phage AB1 (gp15), phage AP22 (gp30), phage phiAC-1 (0043). Phage AB1 (gp27), phage AP22 (gp18), phage phiAC-1 (0031) also share homology to the putative phage head protein encoded by IME-AB2 CDS.24. CDS.71 and CDS.72 of phage IME-AB2 are identified to be associated with tail fiber protein. CDS.74 and CDS.77 of phage IME-AB2 are predicted to encode proteins responsible for baseplate. These two related proteins can be found similar area in the other three phages. The results also demonstrate the phage tail related proteins generally cluster together (Table 3).

### Metabolic proteins

A unique feature of the IME-AB2 genome is that it encodes cobalt transport protein (CDS.6). Notably, cobalt is a cofactor and is required by enzymes from bacteria [15]. It is possible that these metabolic enzymes benefit phage by enhancing the metabolism of the infected bacterial cell, which could in turn increase phage proliferation. No similar cobalt proteins were found in the other three phages sharing homology with IME-AB2. CDS.8 encodes a putative RNA polymerase protein. It is necessary for constructing RNA chains using DNA genes as templates, a process called transcription. Transcription of most double-stranded DNA bacteriophages rely on their host bacteria [16]. The putative CDS.33 of IME-AB2 is predicted to encode HTH domains which have been recruited to a wide range of functions beyond transcription regulation, such as DNA repairing and replication, RNA metabolism and protein-protein interactions in diverse signaling contexts. Beyond their basic role in mediating macromolecular interactions, the HTH domains have also been incorporated into the catalytic domains of diverse enzymes [17]. In functional terms, the HNH endonuclease domain (CDS.52) is found in CRISPR-related proteins. CRISPR functions as a prokaryotic immune system, in that it confers resistance to exogenous genetic elements [18]. CDS.47 is predicted to be a putative bacteriophage-associated immunity protein, which was considered to be responsible for phage superinfection immunity [19]. CDS.68 and CDS.81 are putative lysozyme family protein. Unlike, CDS.0057, 0058, 0059 encoded by phiAC-1, which are putative lysozyme-like domain protein and adjacent to each other, the two lysozyme-like proteins from IME-AB2 are not clustered.

### Packaging-associated proteins

In the four similar *Acinetobacter baumannii* phages, the genetic elements encoding the products involved in the packaging system are commonly found adjacent to one another in the phage genomes. Packaging system is generally composed of big subunit (CDS.27) and small subunit (CDS.28). Usually, the two subunits of the terminase and head portal protein (CDS.26) are closely connected in the packaging system, while the portal protein is a bacteriophage component that forms a hole, or portal, enabling DNA passage during packaging and ejection [20]. It also forms the junction between the phage head (capsid) and the tail proteins.

## Discussion

With the emergence of a growing number of drug-resistant bacterial species, and the difficulties surrounding the development of novel antibiotics [21], exploring novel or alternative therapeutic methods is imperative. The recent renaissance of bacteriophage therapy may provide new treatment strategies for combatting drug-resistant bacterial infections. Although a large number of work on phage therapy in human disease had been done [22-24], the host-specific infection and the relatively narrow lytic spectrum of phage is one of the obstacles to their further application. Individualized phage therapy may represent the future of phage therapy, where bacterial infections will be treated with phage combinations that have already been shown as effective for that particular bacterium. To overcome these limitations, strategies such as screening more lytic phages, combining phages with antibiotics, or administrating phages cocktails should be investigated [25,26]. Therefore, it is very important to isolate novel and sensitive phages to enrich the phage arsenal [27].

All known *Acinetobacter baumannii* bacteriophages were summarized and compared in this research. There are nearly 20 *A. baumannii* phage strains reported in the literature mainly in 2012. Most of the phages genome length are about 40 kb. Just only thirteen complete *A. baumannii* phage genomes were sequenced and deposited in the GenBank database currently. Five of those genomes consist of an approximately 160 kb linear DNA molecule (*Acinetobacter* phage Ac42,Acj61,Acj9,133,and ZZ1), and are annotated as T4-like phage [5,28]. The remaining eight phages contain a genome of 30–50 kb, and may be classified into two different groups according to sequence similarity. In one group, there are four phages with a linear genome, including phiAB1, which was already classified as a ϕKMV-like virus [29], phage YMC/09/02/B1251_ABA_BP [30], *Acinetobacter* phage AB3 and *Acinetobacter* phage Abp1. The other group includes four phages: AB1 (GenBank accession number: HM368260), AP22 (GenBank accession number: HE806280), phiAC-1 (GenBank accession number: JX560521), and our phage IME-AB2. We compared the growth characteristics and the genome of *A. baumannii* phage as shown in Table 4. The results indicated that the newly isolated phage IME-AB2 has a shorter latent and burst period than AB1 and AP22, which implied that the IME-AB2 was more lytic and the burst size produced by IME-AB2 was smaller than those phages [31]. All the listed *A. baumannii* bacteriophages or its lysin had been tested in treating bacterial infection such as inhibiting biofilm formation or effecting on host cell survival. Although the bacteriophage IME-AB2 could lyse the host bacteria and cleared the bacterial suspension in 4 hours, we observed that the resistant bacteria appeared and made the suspension turbid again in 24 hours. The easily emerging resistant bacteria after infection with phage might be an obstacle when fighting against bacterial infection with bacteriophage in the future.

**Table 4:** **Comparative analysis of all known** ***Acinetobacter baumannii*** **bacteriophages**

**Name**	**Morphology**	**Genetic material**	**Genomic length**	**G C content (%)**	**Major protein sizes**	**Adsorption time (>99%)**	**latent period**	**Burst size (PFU/cell)**	**Spectrum**	**Published time**	**Isolation**	**Application**
IME-AB2	Myoviridae	circular dsDNA	43665 bp	37.5	38 kDa (35–130 kDa)	9 min	20 min	62	3 of 22		hospital sewage, Beijing,China	
AB1 [32]	Caudovirales	circular dsDNA	45159 bp	37.7	33.1 kDa (14.4–97.4 kDa)		85 min	232		2012	Wenzhou,China	
AP22 [33]	Myoviridae	circular dsDNA	46387 bp		32 kDa (18–87 kDa)	5 min	40 min	240	89 of 130	2012	Clinical material, Russia	
phiAC-1 [34]	Myoviridae	circular dsDNA	43216 bp	38.5						2012	First phage infect A. soli,South Korea	
AB7-IBB2 [35]	Podoviridae	dsDNA	170 kb			4 min	25 min		19 of 39	2012	India	Inhibit host biofilm formation
AB7-IBB1 [36]	Siphoviridae	dsDNA	75 kb		14.3-43 kDa	5 min	30 min	125	23 of 39	2012	India	Inhibit host biofilm formation
ZZ1 [5]	Myoviridae	linear dsDNA	166682 bp	34.3			9 min	200	3 of 23	2012	fishpond water, Zhenzhou, China	
Abp1 [37]	Podoviridae,phiKMV-like,T7 group	linear dsDNA	42185 bp	39.15	29 kDa–116 kDa		10 min	350	narrow	2012	Chongqing, China	
AB3 [38]	cubic phage	linear dsDNA	31185 bp		35 kDa (35–264 kDa)		20 min	350	wide	2012	Chongqing, China	
YMC/09/ 02/B1251 ABA BP [30]	Podoviridae	linear dsDNA	45364 bp	39.05						2012	South Korea	
phikm18p [39]	cubic phage	dsDNA	45 kb		39 kDa				wide	2012	Taiwan	Cell survival test
ABp53 [40]	Myoviridae	dsDNA	95 kb		47-kDa protein		10 min	150	27%	2011	Sputum,Taiwan	
phiAB1 [29]	Podoviridae, phiKMV-like phages	linear dsDNA	41526 bp							2011	Taiwan	
Ac42,Acj61,Acj9,133 [28]	T4	linear dsDNA	160 kb							2010	USA	
AB1 [41]	Siphoviridae family	dsDNA	45.2 kb to 46.9 kb		14 to 80 kilo-dalton		18 min	409	narrow	2010	Marine sediment, Taiwan	
phi AB2 [42]	Podoviridae, phiKMV-like phages	dsDNA	40 kb			8 min	10 min	200	wide	2010	Taiwan	Lyase AB2

## Conclusions

A lytic *A. baumannii* bacteriophage IME-AB2 was isolated and characterized in this research. The complete genome of IME-AB2 was sequenced and compared to those of *A. baumannii* phage AB1, *A. baumannii* phage AP22, and A. *baumannii* phage phiAC-1 in detail. The genome of IME-AB2 was replete with novel genes without known relatives, which indicated that IME-AB2 was a novel and unique *A. baumannii* bacteriophage. Although the resistant *A. baumannii* appeared finally after infection with IME-AB2, the comprehensive understanding of the phage’s characteristics is conducive to the treatment of multidrug-resistant *A. baumannii* in the future.

## Methods

### Bacterial strains, Phage isolation, propagation, and titration

This study included 22 clinical strains of *A. baumannii* (MDR-AB1139, MDR-AB1, MDR-AB2, MDR-AB3, MDR-AB4, MDR-AB5, … , MDR-AB19,MDR-AB20, MDR-AB21). All the clinical samples were taken as part of standard patient care at the PLA Hospital 307, Beijing, China. The patients were orally informed that the specimens would be used for screening bacteria and the tests were optional on laboratory sheet. Blood, sputum and skin swabs were collected from patients with consent under the Ethics Committee of the PLA Hospital 307. The protocol of screening bacteria was approved by the Ethics Committee of the PLA Hospital 307 and Beijing Institute of Microbiology and Epidemiology Ethics Committee.

Multidrug-resistant *A. baumannii* strain MDR-AB2 was used as an indicator for bacteriophage screening of raw sewage samples collected from PLA Hospital 307. Sewage samples were separated by centrifugation at 12,000 × *g* for 20 min. Following removal of the solid impurities by centrifugation, the supernatants were filtered through a 0.45 μm pore-size membrane filter to remove bacterial debris. Filtrate (4 ml) was added to 2 ml of 3× Luria-Bertani (LB) broth medium and mixed with 0.1 ml of *A. baumannii* overnight culture (OD_600_ = 0.6) to enrich the phage at 37°C overnight. Following enrichment, the culture was centrifuged at 12,000 × *g* for 10 min, and then the supernatant was filtered with a 0.45 μm pore-size membrane filter to remove the residual bacterial cells. The filtrate (0.1 ml) was mixed with 0.5 ml of *A. baumannii* in LB culture (OD_600_ = 0.6) and 5 ml of molten top soft nutrient agar (0.75% agar), which was then overlaid onto solidified base nutrient agar (1.5% agar) [43]. Following incubation for 6 h at 37°C, the clear phage plaques were picked from the plate. The phage titer was determined using the double-layered method previously described by Adams [44].

### Phage concentration , purification and storage

A single plaque was picked into 5 ml of LB medium containing MDR-AB2 (OD_600_ = 0.6) and cultured at 37°C for 6 h. A 5 ml aliquot of suspension was transferred into 500 ml of LB medium for culture at 37°C overnight. Chloroform was then added to the 500 ml of culture to a final concentration 0.1% before being mixed gently and allowed to stand at room temperature for about 30 min. Solid NaCl was added to the culture to a final concentration of 1 M, which was then incubated in an ice water bath for 1 h. The culture was centrifuged at 11,000 × *g* for 10 min to remove cell debris, and polyethylene glycol 6000 (PEG6000) was added to the supernatant to a final concentration of 10% (w/v) while slowly stirring with a magnetic stirrer at room temperature. This solution was transferred to a polypropylene centrifuge tube in an ice water bath and incubated at least 1 h to precipitate the phage particles. Following centrifugation (11,000 × *g* for 10 min at 4°C), the phage-containing precipitate was resuspended in 5 ml of SM buffer (50 mM Tris-Cl, 100 mM NaCl, 8 mM MgSO_4_, pH 7.5) [45]. An equal volume of chloroform was then added to separate the phage particles from PEG6000. Following centrifugation at 3,000 × *g* for 10 min, the aqueous phase was recovered and filtered through a 0.22 μm pore-size membrane filter to remove debris. The concentrated 1.0 ml of phage suspensions were layered on the top of a cesium chloride gradient solutions (density of 1.3 g/ml-0.45 g of cesium chloride in 1.0 ml of water; density of 1.5 g/ml-0.83 g of cesium chloride in 1.0 ml of water; density of 1.7 g/ml-1.28 g of cesium chloride in 1.0 ml of water) in 5.0 ml cellulose nitrate centrifuge tube [46]. After centrifugation in a Beckman Coulter Swinging Bucket Rotor (SW41, Ti) for 40 min at 100,000 × g, the concentrated phages at the visible band were collected by means of a capillary pipette. The purified phage was stored at 4°C.

### Determination of lytic spectrum

The host range was determined by spot test. Briefly, 0.5 ml of bacterial overnight culture was mixed with 5 ml of molten top soft nutrient agar (0.75% agar) and then overlaid on the surface of solidified base nutrient agar (1.5% agar). Once the top layer also solidified, 2 μl of the phage preparation(1 × 10^9^ pfu/ml) was spotted onto the plate, which was incubated for 6 h at 37°C.

### Electron microscopy

Phage stock solution was directly stained with phosphotungstic acid (PTA) for 2 min. After being dried at room temperature, the grid was examined using a Philips TECNAI-10 transmission electron microscope (TEM) to observe and record the morphology of the phage particles [41].

### Extraction of phage genomic DNA

Purified phage particles were treated with DNase I (1 μg/ml) (Takara) and RNase A (1 μg/ml) (Takara) for 30 min, and then the nucleases were inactivated at 80°C for 15 min. Ethylene diamine tetraacetic acid (EDTA) (20 mM), proteinase K (50 μg/ml) and sodium dodecyl sulfate (SDS) (0.5%) were then added and the mixture was incubated at 56°C for 1 h. Phage lysate was extracted with an equal volume of phenol:chloroform:isoamyl alcohol (25:24:1). Chloroform extraction was repeated until there was no phenol odor. An equal volume of isopropanol (AR grade) was added and the sample was incubated overnight at -20°C to precipitate the phage genomic DNA. The pellet was washed with 75% ethanol, and then deionized water was used to dissolve the precipitated genomic DNA.

### Whole genome sequence and bioinformatics analysis

The genomic DNA of IME-AB2 was subjected to high-throughput sequencing using a Life Technologies Ion Personal Genome Machine Ion Torrent sequencer (San Francisco, CA) according to the manufacturer’s instructions. The complete genome sequence of phage IME-AB2 was assembled using Velvet [47] and CLC Bio (Aarhus, Denmark), and annotated using RAST [48] and InterPro [49]. Sequence similarity analysis and comparison were performed using NCBI packages.

## Competing interests

The authors declare that they have no competing interests.

## Authors’ contributions

Fan Peng isolated the phage, identified the characterization, and drafted the manuscript. Zhiqiang Mi and Yong Huang were responsible for sequencing and drafting the manuscript. Wenkai Niu and Xin Yuan collected clinical bacteria. Yahui Wang conducted the phage concentration and purification. Yuhui Hua and Huahao Fan conducted EM assay. Yigang Tong and Changqing Bai conceived and designed the experiments. All authors read and approved the final manuscript.
